# An evaluation of the PCR-RFLP technique to aid molecular-based monitoring of felids and canids in India

**DOI:** 10.1186/1756-0500-3-159

**Published:** 2010-06-07

**Authors:** Shomita Mukherjee, Ashalakshmi CN, Chandrima Home, Uma Ramakrishnan

**Affiliations:** 1 National Centre for Biological Sciences, GKVK Campus, Bellary Road, Bangalore 560 065, Karnataka, India

## Abstract

**Background:**

The order Carnivora is well represented in India, with 58 of the 250 species found globally, occurring here. However, small carnivores figure very poorly in research and conservation policies in India. This is mainly due to the dearth of tested and standardized techniques that are both cost effective and conducive to small carnivore studies in the field. In this paper we present a non-invasive genetic technique standardized for the study of Indian felids and canids with the use of PCR amplification and restriction enzyme digestion of scat collected in the field.

**Findings:**

Using existing sequences of felids and canids from GenBank, we designed primers from the 16S rRNA region of the mitochondrial genome and tested these on ten species of felids and five canids. We selected restriction enzymes that would cut the selected region differentially for various species within each family. We produced a restriction digestion profile for the potential differentiation of species based on fragment patterns. To test our technique, we used felid PCR primers on scats collected from various habitats in India, representing varied environmental conditions. Amplification success with field collected scats was 52%, while 86% of the products used for restriction digestion could be accurately assigned to species. We verified this through sequencing. A comparison of costs across the various techniques currently used for scat assignment showed that this technique was the most practical and cost effective.

**Conclusions:**

The species-specific key developed in this paper provides a means for detailed investigations in the future that focus on elusive carnivores in India and this approach provides a model for other studies in areas of Asia where many small carnivores co-occur.

## Introduction

India is inhabited by 58 of the 250 globally distributed species of carnivore. This is especially striking in the Family Felidae where 15 of the 36 species inhabit India, making it the richest in worldwide cat diversity [1]. Although many studies have been initiated on larger carnivores such as the lion [2], tiger, leopard and wild dog [3-5], snow leopard [6,7] and wolf [8], knowledge about the distribution, ecology and conservation status of many of the smaller carnivores remains poor. This is largely due to their rare, elusive and nocturnal habits, coupled with cumbersome bureaucratic formalities involved in invasive studies on rare species. As a result, few detailed ecological studies exist on small-carnivores in India [9,10].

Traditional non-invasive field studies, based on indirect evidence from tracks and scats, have great potential for the study of these small carnivore species. However, because of the co-occurrence of several small carnivores, accurate species-level assignments are mandatory especially for studies designed to determine species presence, diet and many other aspects of their ecology. Discriminating between scats of various species can be done using laboratory techniques like thin-layer-chromatography (TLC) [11]. However, an error of up to 29% was demonstrated in species identification using TLC [12]. Moreover, TLC can only be used for species identification and estimates of relative abundance, limiting its utility compared to faecal genetic typing information on both gender and species. Molecular approaches [13-17] provide a more accurate and standardizable alternative to TLC. Such approaches include use of species-specific primers [17,18], melting curve analysis [16] and Polymerase Chain Reaction (PCR)-Restricted Fragment Length Analysis (RFLP) [15,19-22]. The former two approaches are less effective when several closely related species live in sympatry. The latter approach, PCR-RFLP (where a PCR with common primers is followed by restriction digestions targeting species specific motifs) has been used to survey carnivores from faeces. Thus far, this approach has been used to distinguish between a maximum of 7 sympatric mammalian carnivore species [15,23]. However, not all scats yield results, and amplification success/failure depends on the degree of degradation of the scat [23]. Because different geographic regions would have varied climatic conditions, we expect the success of scat based molecular analysis to vary among regions.

Due to the vast diversity of carnivores in India, many occur in sympatry, and hence a protocol for assigning scats accurately to species is urgently required for initiating any study on the group. In this paper, we develop and test a set of protocols for identifying Indian felids and canids based on the PCR-RFLP technique applied to DNA extracted from faecal samples. India includes a diverse set of ecosystems representing varied climatic conditions. Therefore, we investigated the applicability of our methods to scat samples collected from various biogeographic zones [24] within India. Finally, we discuss the utility of our non-invasive molecular tools in the context of surveying small carnivores in India and Asia.

## Methods and Results

### Standardization and validation of PCR-RFLP panel

Selected GenBank (Table 1) entries of mitochondrial 16S rRNA sequences from Indian carnivores were aligned in MEGA 4.0 [25] and used to design the following PCR primers:

**Table 1:** DNA fragment profile (expected and observed) after amplification with Felid 16S rRNA primers and restriction digestion.

Species	GenBank Accession numbers	Taq I	Hae III	Ase I	Nla III	Pac I	Dpn I
Jungle cat (*Felis chaus*)	AF006393.1	1	2	2	1	3	1

Caracal (*Caracal caracal*)	AF006389.1	1	2	1	1	1	1

Leopard cat (*Prionailurus bengalensis*)	AF006437.1	2	1	2	2	2	1

Fishing cat (*Prionailurus viverrinus*)	AF006451.1	2	1	2	1	2	1

Rusty-spotted cat (*Prionailurus rubiginosus*)	AF006445.1	1 (2)*	2 (1)*	1 (2)*	1	2	1

Lynx (*Lynx lynx*)	AF006413.1 AY499288.1 AY499289.1 AY499290.1 AY499291.1	2	1	1	1	2	2

Manul (*Otocolobus manul*)	AF006431.1	1	2	1	2	2	1

Marbled cat (*Pardofelis marmorata*)	AY499300.1 AF006439.1	1	2	1	1-2^#^	2	1

Asian golden cat (*Catopuma temminkii*)	AF006447.1	1	2	1	1	2	2

Domestic cat (*Felis silvestris catus*)	AF006453.1	1	2	1	1	2	1

Wild cat grp. (*Felis silvestris*)	AF006395.1 AF006401.1	1	2	1	1	2	1

Clouded leopard (*Neofelis nebulosa*)	AF006425.1 AY499301.1	2	1	1	1-2^# ^(2)*	2	2

Lion (*Panthera leo)*	AF006457.1 FJ151641.1 FJ151644.1 FJ151652.1	2	1	1	2	2	2

Tiger (*Panthera tigris*)	EF394928.1 EF392683.1 EF551003.1 AY452110.1	2	2	2	2	2	1

Leopard (*Panthera pardus*)	AF006443.1 DQ904388.1 EU223367.1	2	1	1	1-2^# ^(2)*	2	3

Snow leopard (*Uncia uncia*)	AF006449.1 EF551004.1	1	1	1	2	2	2

* Figures in brackets indicate our results that were different from what was expected.

# Two or more sequences from GenBank with differing restriction digestion patterns.

1) Felid 16S rRNA F: 5' GCTCTACTGTCTCTTACT 3' and Felid 16S rRNA R: 5' TCAAATCACTCGGAGGTT 3'

2) Canid 16S rRNA F: 5' ACTGTCTCTTACTCCCAA 3' and Canid 16S rRNA R: 5' TTATATTCCGAGGTCACC 3'.

This allowed for the amplification of a region of 210 bp for felids (location of segment on *Felis silvestris catus *mitochondrial DNA: 2946 bp to 3156 bp) and 189 bp for canids (location of segment on *Canis lupus *mitochondrial DNA: 2079 bp to 2268 bp). The primers were designed and tested (standardization was conducted with DNA from blood of domestic cat and dog) with both negative controls and nucleotide sequencing of amplification products, in an effort to verify the specificity of amplification products. To standardise PCR conditions for the primers, we used blood samples of house cat and domestic dog. The PCR master mix included the premixed Taq DNA polymerase, buffer and dNTPs (QIAGEN, Inc.), 4 μg Bovine Serum Albumin (Sigma) and 2 μM primer for 10 μl PCR reactions. Conditions of amplification included initial denaturation at 94°C for 10 min, followed by 59 cycles of denaturation at 94°C for 30 s, annealing at 40°C-52°C for 45 s and cycle extension at 72°C for 50 s and a final extension at 72°C for 10 minutes. The PCR products were visualized in a 2% agarose gel. The best annealing temperatures were determined to be 49°C for the felid primers and 48°C for the canid primers. Since the primers were developed around conserved regions, we had to rule out the possibility of amplifying prey DNA. Therefore, we sequenced amplified products from some field-collected scats of known identity that contained prey remains of various taxa and species.

We used a web based program, NEB cutter V.2 [26], to select restriction enzymes that would differentially cut the DNA fragments for each species and prepared a chart on potential differentiation of felid and canid species using several enzymes (with the number of fragments produced after digestion) (Table 1, 2). We then selected the minimum number of enzymes that would resolve a maximum number of species within each family. For the GenBank sequences, we were able to distinguish between all Indian canids and felids based on a set of eight restriction enzymes (*Taq *I, *Hae *III, *Ase *I, *Nla *III, *Pac *I, *Xsp*1/*Bfa *I, *Bsm *I, *Ssp *I). Tables 1 and 2 reveal the predicted restriction digestion pattern given by these enzymes.

We obtained scat samples from captive individuals of 10 species of cats (house cat, jungle cat, leopard cat, fishing cat, rusty-spotted cat, golden cat, snow leopard, clouded leopard, leopard, and tiger) and five species of canids (domestic dog/Indian wolf/Himalayan wolf, Asiatic wild dog, golden jackal, desert fox, Bengal fox) from captivity, and tested the predicted patterns for these species. We extracted DNA using QIAmp (QIAGEN) tissue and stool kits following the manufacturer's protocols with slight modifications [18]. We amplified the desired region in a 15 μl reaction annealing temperatures of 49°C for felids and 48°C for canids. The 16S rRNA primer sets worked with all 10 species of felids and five species of canids.

Restriction enzyme incubations were set for 10 μl reactions with 1 μl of 10× buffer, 1 μl of enzyme (5 U), and 0.5-1.5 μl of PCR product (depending on the intensity of the band after amplification). 1 μl of BSA (4 μg) was added according to manufacturer's instruction for the enzymes *Taq *I, *Nla *III and *Pac *I. Water (Milli-Q) was used to make up the rest of the volume for the reaction. Incubations were carried out for 3 hours at 35°C for all enzymes except *Taq *I and *Bsm *I which were incubated at 65°C. Fragments were visualised in a 2% agarose gel.

**Figure 1:**
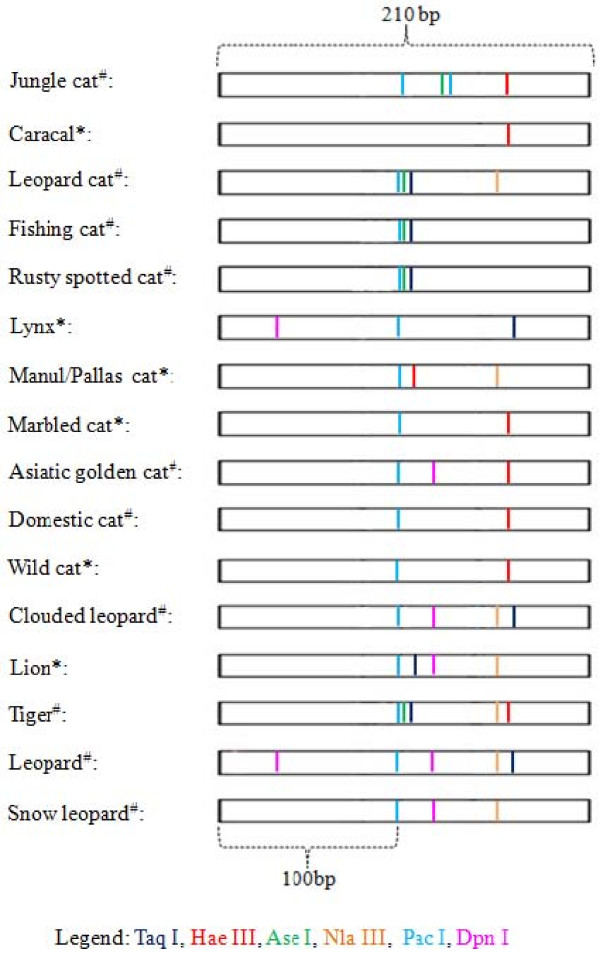
**Restriction digestion profile for felids**. *Expected from available sequence. ^#^Observed from this study for 10 species of felids after amplification with Felid 16S rRNA.

Enzyme digestions produced expected results in all felids except the rusty-spotted cat where the predicted pattern for the enzymes *Taq *I, *Hae *III and *Ase *I were not as expected (Table 1, Figure 1).

For the canids, patterns for golden jackal, desert fox and dhole were as expected, but the wolf-domestic dog group (domestic dog, Indian wolf, and Himalayan wolf) showed patterns that differed from what was predicted (Table 2, Figure 2). We tested this with scats of other individuals of the same species and our results were confirmed. Additionally, sequencing confirmed the difference between the GenBank records and the ones generated in our laboratory. Our results revealed that a combination of 3 enzymes maximally differentiated within felids and canids. (Figures 1, 2).

**Table 2:** DNA fragment profile (expected and observed) after amplification with Canid 16S rRNA primers and restriction digestion.

Species	GenBank Accession numbers	Bsm I	Xsp1/Bfa I	Ssp 1	Ase I	Nla III
Domestic dog (*Canis lupus familiaris*)	EU789728.1 EU789759.1 EU789784.1	1	2	1	1	1 (2)*

Gray wolf (*Canis lupus*)	AM711902.1 DQ480507.1 DQ480508.1	1	2	1	1	1-2^# ^(1)*

Himalayan wolf (*Canis lupus chanco*)	EU442884.2 AY289963.1 GQ374438.1	1	1-2^# ^(1)*	1	1	1

Golden jackal (*Canis aureus*)	AY289969.1 AY289970.1	2	1	1	1	1

Asiatic Wild dog (*Cuon alpinus*)	AY289971.1 AY289972.1 GU063864.1	1	1	2	1	1

Red fox (*Vulpes vulpes*)	AM181037.1 GQ374180.1 DQ334815.1	1	1	1	2	1

Bengal fox (*Vulpes bengalensis*)		(1)		(2)	(2)	(1)

* Figures in brackets indicate our results that were different from what was expected.

# Two or more sequences from GenBank with differing restriction digestion patterns.

**Figure 2:**
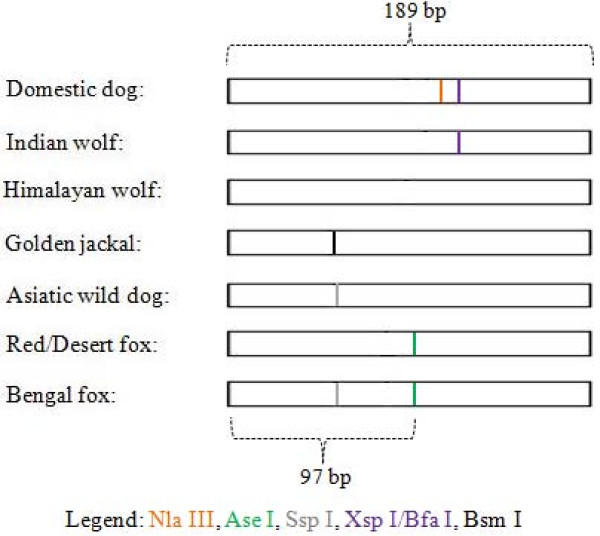
**DNA fragment profile (observed) after amplification with Canid 16S rRNA primers and restriction digestion**.

### Applying protocols under field conditions

We tested our protocols on scats collected from natural habitats because field collected scats are subjected to a variety of environmental conditions. We tested protocols only for the felid panel. Scats were collected from various parts of the country, representing major biogeographic regions [24]. We selected scats for testing that were visually assigned to felids. We extracted DNA from 131 scats, representing four biogeographic zones (66 scats from the arid zone/Thar desert, 27 from the Western-Ghats, 21 from the semi-arid zone, and 17 from the Himalayas). In order to reduce costs, we modified the extraction protocol and replaced the 'InhibitEX' tablets provided in the Qiagen stool kit with 600 mg of starch and 600 μg of BSA [27].

**Figure 3:**
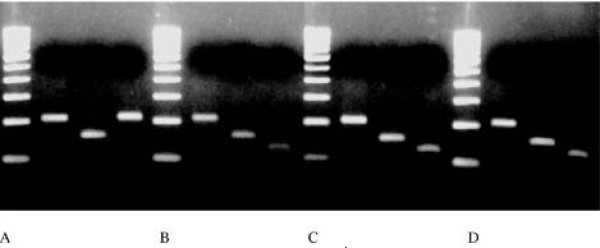
**Some results of the test of the technique on scats collected in natural habitats**. Gels include a 100 bp Ladder; Enzymes used from left to right: Taq I, Hae III, Ase I. A: House cat; B, C and D: Jungle cat.

Amplification success, using scats from natural habitats, was 52% (Table 3). We used 56 of these samples for restriction digestion (Table 3). Of these, 86% (48 scats) could be assigned to single species (jungle cat, leopard cat, house cat/Asiatic wildcat, rusty spotted cat and snow leopard), while 14% remained unresolved (Table 3, Figure 3). The results were confirmed through sequencing. We did not sequence products that were not resolved by restriction digestion.

**Table 3:** Results of the PCR-RFLP technique on scats collected from various biogeographic zones in India.

	Arid	Semi-arid	Western-Ghats	Himalayas	Total
DNA extraction	66	21	27	17	131

Amplification success (%)	33 (50)	14 (67)	14 (52)	8 (47)	68 (52)

RFLP	24	11	14	7	56

Jungle cat	12	10	3	0	25

HC/WC	8	0	3	0	11

Leopard cat	0	0	4	6	10

Rusty-spotted cat	0	0	1	0	1

Snow leopard	0	0	0	1	1

Unresolved	4	1	3	0	8

Finally a comparison of costs across the various techniques currently used for scat assignment showed that the PCR-RFLP technique was the most cost effective (Table 4).

**Table 4:** A comparison of cost per sample for various molecular scat identification techniques, in INR, GBP and USD.

Method	Extraction (starch)	PCR	Sequencing	RFLP (3 enzymes)	Total
Specific	150.00	50.00		-	200.00
Primers	2.00	0.70			2.70
	3.30	1.10			4.40

Sequencing	150.00	50.00	400.00	-	600.00
	.00	0.70	5.30		8.00
	3.30	1.10	8.90		13.30

RFLP	150.00	50.00	-	50.00	250.00
	2.00	0.70		0.70	3.40
	3.30	1.10		1.10	5.50

## Discussion

For some felids and canids, our results of enzyme digestion produced patterns that were different from expected based on sequences from GenBank. In some cases where sequences were available for two or more individuals of the same species polymorphism was seen, which resulted in more than one restriction digestion pattern for the species (Table 1). These could be due to true polymorphism or sequencing errors. We ruled out sequencing errors in our study by repeated sequencing from the same as well as different individuals of a given species.

In some cases polymorphism within a species can lead to errors in identification, unless the populations/subspecies are geographically isolated and there is no polymorphism within each population. The wolf sequences available on GenBank were of *C. lupus lupus *and *C. l. chanco*, and not *C. l. pallipes *(Indian wolf) and the digestion patterns did not match. Similarly the rusty spotted cat sequence on GenBank is perhaps from Sri Lanka. These explanations need to be verified with additional sequences from individuals within populations as well as from individuals across different populations of each species. On the other hand, the house cat (*Felis silvestris catus*) and Asiatic wildcat (*Felis silvestris ornata*) are closely related, occur together and have identical sequences for the 16S rRNA region that we used for the RFLP. Since these two cats can potentially hybridize, we suggest the use of nuclear markers for further resolution. Furthermore, hybridisation between closely related species of felids is known to occur, and this is a potential drawback of a technique using only mtDNA [15]. More studies at the population level for each species are required to determine the degree of hybridisation and the error associated with surveys restricted to the use of mtDNA markers.

Although some felid species have identical digestion patterns (e.g. Asiatic lion and clouded leopard), their habitats never overlap and so there should be no confusion. However, species identities that could not be fully resolved with our panel of enzymes (clouded-leopard/leopard) and can occur sympatrically need to be examined using other genes and enzymes.

A proportion of field samples and some of the control samples revealed ambiguous results. This could be because PCR products were only partially digested. In such cases, we repeated the digestion with fresh reagents and a lower concentration of PCR product in the reaction. It is difficult to suggest a standard amount of PCR product to use in a reaction due to variation in DNA quantity after amplification from different scats. We, however, recommend a rough standardization by visually determining the DNA concentration of the PCR product through band intensity, and adjusting its volume in the reaction. For example, in our case we loaded 1 μl of ladder that provided a DNA concentration of 50 ng for the 200 bp band. If the band intensity was equal to or half that of the ladder, we used 0.5 μl of the product in the digestion mixture, whereas for bands less than half the intensity of the ladder, we used 1.5 μl of PR product. Our results revealed a lower number of unresolved samples when we followed this protocol.

Other factors that could lead to errors in assignment are amplification of DNA of species ingested by the predator. Although we tested for prey DNA and found that our primers specifically amplified carnivore DNA, there is also a possibility of the carnivore preying on another carnivore. In such cases it may result in confusing restriction patterns or an error in assignment. We however presume that such cases will be rare.

One reason for the relatively low amplification success for field-collected scats could be incorrect field identification of scats as felid. To exclude this possibility, scats first need to be tested with primers for canids and other carnivores. Alternatively, the scats could be old at the time of collection, or subjected to adverse environmental conditions like sunlight or water, both of which would result in low quantity and quality of DNA.

Degradation time for scats in various biogeographic zones is likely to differ, with the wetter zones such as the Western-Ghats and Himalayas having a larger proportion of degraded scats. Our data do reveal that environmental conditions impact amplification success. Habitats with high rainfall and temperature showed lower amplification success. Perhaps in such areas the scat collection success itself will be poor. On the other hand, hot and cold deserts (the Thar and high altitude Himalayas) are also likely to have scats with degraded DNA due to exposure to UV rays, even if the scats do not appear to be degraded. The semi-arid region, constituting a large part of India revealed the highest species identification success. These results also point to the importance of the timing of surveys for scat collection. Our experience suggests that through most of India the best period for scat collection is from November to May.

## Conclusions

This was the first time in India that molecular techniques have been standardised for carnivore scat identification. One of the scats collected from the BR Hills sanctuary in the Western-Ghats in Southern India turned out to be of the rusty spotted cat. This was the first record of this cat for the sanctuary and is an example of the value of this technique in surveying small elusive carnivores, including those like the rusty spotted cat, whose distributions are till now ambiguous. Furthermore, it also demonstrates how integral molecular tools have become to ecology in current times. The type of key we develop in this paper paves the way for detailed investigations on elusive carnivores in India in the future, using an efficient and relatively cheap technique.

## Competing interests

The authors declare that they have no competing interests.

## Authors' contributions

SM conceived and designed the study, raised grants, participated in coordination, sample collection, primer design, data generation and analysis (extractions, PCR, RFLP) interpretation of data and writing of manuscript. ACN helped with data generation and analysis, interpretation of data and drafting of manuscript. CH helped with primer design, sample collection, data generation and analysis, interpretation of data, troubleshooting with techniques and revising manuscript. UR helped with study design, raising grants, coordination, discussions and revising manuscript.
